# RNAi-based validation of antibodies for reverse phase protein arrays

**DOI:** 10.1186/1477-5956-8-69

**Published:** 2010-12-23

**Authors:** Heiko A Mannsperger, Stefan Uhlmann, Christian Schmidt, Stefan Wiemann, Özgür Sahin, Ulrike Korf

**Affiliations:** 1 German Cancer Research Center, Division of Molecular Genome Analysis, Heidelberg, Im Neuenheimer Feld 580, 69120 Heidelberg, Germany

## Abstract

**Background:**

Reverse phase protein arrays (RPPA) have been demonstrated to be a useful experimental platform for quantitative protein profiling in a high-throughput format. Target protein detection relies on the readout obtained from a single detection antibody. For this reason, antibody specificity is a key factor for RPPA. RNAi allows the specific knockdown of a target protein in complex samples and was therefore examined for its utility to assess antibody performance for RPPA applications.

**Results:**

To proof the feasibility of our strategy, two different anti-EGFR antibodies were compared by RPPA. Both detected the knockdown of EGFR but at a different rate. Western blot data were used to identify the most reliable antibody. The RNAi approach was also used to characterize commercial anti-STAT3 antibodies. Out of ten tested anti-STAT3 antibodies, four antibodies detected the STAT3-knockdown at 80-85%, and the most sensitive anti-STAT3 antibody was identified by comparing detection limits. Thus, the use of RNAi for RPPA antibody validation was demonstrated to be a stringent approach to identify highly specific and highly sensitive antibodies. Furthermore, the RNAi/RPPA strategy is also useful for the validation of isoform-specific antibodies as shown for the identification of AKT1/AKT2 and CCND1/CCND3-specific antibodies.

**Conclusions:**

RNAi is a valuable tool for the identification of very specific and highly sensitive antibodies, and is therefore especially useful for the validation of RPPA-suitable detection antibodies. On the other hand, when a set of well-characterized RPPA-antibodies is available, large-scale RNAi experiments analyzed by RPPA might deliver useful information for network reconstruction.

## Background

### Reverse phase protein arrays

The potential use of the RPPA technology in the field of proteomics and systems biology was introduced in 2001 by Paweletz and colleagues [1]. Since then, the RPPA technology has been further advanced, and successfully applied in numerous proteomic studies [2-13]. The basic principle of RPPA follows the idea of a dot-immunoblot; large numbers of samples are arrayed on solid phase carriers, and each array can then be probed with a different highly specific antibody. RPPA provide a semi-quantitative readout, and the expression of a particular target protein can be compared among all samples printed on a particular array. Printing of numerous replicate slides permits access to a highly parallelized analysis since each slide can be probed with a different detection antibody.

### Characterization of antibody specificity for RPPA applications

The outcome of all types of immunoassays strongly depends on antibody specificity and affinity. These factors are far more important for RPPA compared to other immunoassays such as Western blotting (WB), immunohistochemistry (IHC) or sandwich ELISA/microspot immunoassay (MIA). In detail, in Western blot experiments, unspecific binding of antibodies can frequently be identified by taking into account the reported molecular weight (MW) of a certain target protein. Similarly, in IHC experiments, antibody cross-reactivity can be identified by paying attention to the expected cellular or sub-cellular localization of a certain target protein. For antibodies used in sandwich MIA and in ELISA, a slight cross reactivity can be tolerated if the off-target binding properties of the two antibodies do not overlap. In contrast, antibody specificity for RPPA experimentation has to be assessed separately and beforehand the RPPA analysis. Characterizing antibodies by Western blot is a commonly accepted strategy [1,7,14]. However, even those antibodies showing mono-specificity on Western blot as well as a linear correlation between the signal intensity and the corresponding dilution step of serially diluted samples do not always quantify the corresponding target proteins correctly. Even minor unspecific binding contributes to the signal of a certain spot on RPPA. Furthermore, since RPPA are a high-throughput tool with totally or partially automated incubation protocols, it is not possible to optimize the incubation conditions for individual antibodies as common in Western blot strategies, and experimental conditions are chosen to work for the majority of RPPA antibodies. Interactions between an epitope of a target protein and the corresponding paratope of the antibody are influenced by small experimental changes of pH, temperature, ion concentration or detergents. Similarly, target protein conformation, sample pretreatment, and composition of the protein matrix influence the interaction between an epitope and its paratope. Considering all these parameters, it is comprehensible that Western blot results do not necessarily correspond with RPPA outcome. Nevertheless, Western blot will remain an indispensable tool for the characterization of antibody specificity, and is also used for antibody validation in the approach introduced here.

### RNA interference

RNA interference (RNAi) is a biological process where small RNA molecules silence gene expression, either by inducing sequence specific degradation of target mRNA or by inhibiting translation [15]. After its first discovery by Fire and Mello in *C. elegans *[16] and the proof that this mechanism can be exploited for the manipulation of mammalian cells [17], RNAi opened up a new era in reverse genetics and enabled large-scale loss-of-function studies. Chemically synthesized small interfering RNA (siRNA) molecules have been shown to be potent effectors of post-transcriptional gene silencing and result in the specific inhibition of protein expression. For this reason, RNAi is considered as one of the most promising tools to dissect biological processes. We have previously applied RPPA to quantify protein expression after applying multiple siRNAs simultaneously [18], as well as to reconstruct protein networks by quantifying proteins of a given network after a knockdown of selected proteins [19,20].

### The potential of RNAi to validate antibodies for RPPA applications

Positive controls for the validation of phospho-specific antibodies can easily be generated by treating a cell line with UV light or growth factors [21]. By this way, suitable controls can be generated to discriminate between phosphorylated and non-phosphorylated forms of a certain protein. However, positive controls for general target protein-specific antibodies require either the elimination of a certain protein from a complex mixture or the introduction of the protein of choice into a suitable matrix to mimic the biological context. Likewise, this strategy can also be applied to characterize protein isoform-specific antibodies. Moreover, the limit of detection (LOD) might be of interest for quantitative RPPA applications which can be determined by using a spike-in approach. However, when performed on a larger scale for numerous targets the spike-in strategy would require access to large numbers of purified and well-characterized proteins. Recombinant proteins are frequently tedious to obtain and heterologously produced recombinant proteins are not necessarily recognized by antibodies with an affinity and specificity comparable to endogenous protein. An alternative strategy might be RNAi; first, to validate the specificity of antibodies and, secondly, to determine the LOD of specific antibodies. For this reason, serial dilutions of siRNA-treated samples and untreated samples as controls were printed in parallel onto nitrocellulose-coated slides which were probed with a panel of different detection antibodies directed against the target protein of choice. In addition, the knockdown efficiency was determined in parallel by Western blot. Antibodies which detected the reduced abundance of the targeted protein in siRNA-treated samples and with a rate comparable to that measured by Western blot were considered as suitable for use in RPPA applications.

## Results

### Antibody validation using quantitative Western blot

**Figure 1:**
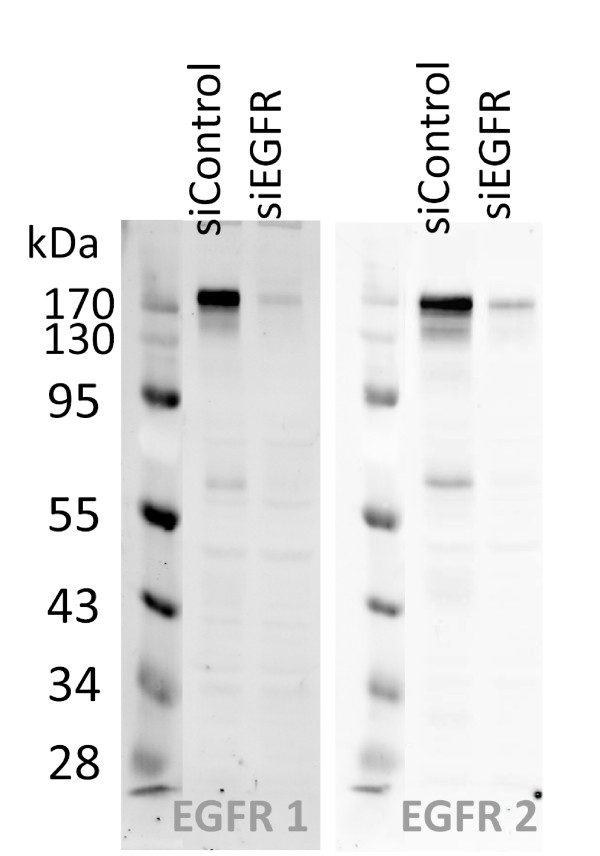
**Target specificity of anti-EGFR antibodies**. Two different anti-EGFR antibodies were compared by Western blot to detect endogenous EGFR in MDA-MB-231 cell lysate before and after siRNA-induced knockdown.

**Figure 2:**
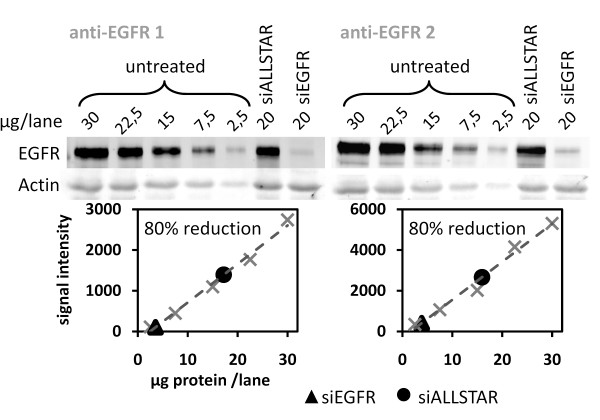
**Knockdown detection capacity of anti-EGFR antibodies**. EGFR levels of siALLSTAR and siEGFR transfected MDA-MB-231 cells were quantified using two anti-EGFR antibodies and a linear regression model fitted on a serially diluted MDA-MB-231 cell lysate. Both antibodies showed identical readout for Western Blot-based knockdown quantification.

Antibody specificity was initially assessed by Western blot. For example, figure 1 shows the detection of EGFR in MDA-MB-231 cell lysates by two different anti-EGFR antibodies. In detail, both antibodies recognized the predominant band of (~175 kDa) molecular weight, corresponding to the reported size of EGFR, and a weak low molecular weight band of approximately 60 kDa. Next, EGFR was knocked-down by RNAi in MDA-MB-231 cells and quantified by Western Blot (figure 2). To calculate the knockdown rate, a linear regression was calculated on the median of a five-step MDA-MB-231 dilution series, and slope and intercept were used to determine the relative concentration of EGFR protein in the positive controls as well as in the siEGFR transfected samples. Both antibodies indicated that EGFR abundance was reduced by 80% as a result of EGFR silencing. Figure 2 illustrates that specificity and quantitative data obtained for both anti-EGFR antibodies by Western blot were comparable. However, the EGFR signal obtained with antibody 2 was twice higher compared to that after probing with antibody 1.

### Antibody validation on RPPA

**Figure 3:**
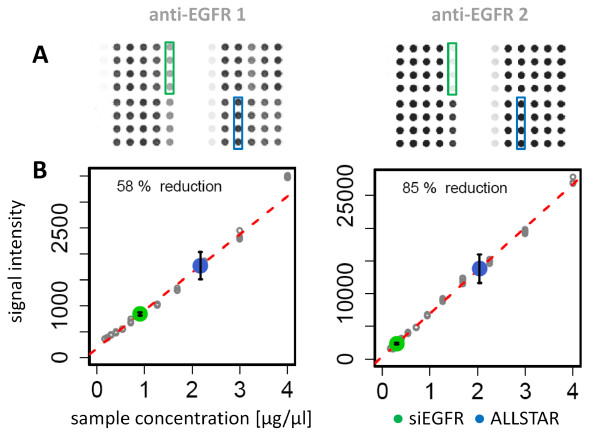
**RPPA quantification of EGFR knockdown**. Two biological samples and four control samples were printed on nitrocellulose coated slides (four technical replicates per sample). Signal detection was performed using NIR-fluorescent dye labeled secondary antibodies. Position of siEGFR (green) and siALLSTAR (blue) transfected samples is indicated (A). Spots are false color images of fluorescent signals. None of the signals reached the saturation range of the scanning instrument (Odyssey, LI-COR). Target protein knockdown was determined by comparing EGFR signal intensity between siEGFR and siAllstar transfected samples. The linear regression was calculated from a dilution series of whole cell lysate from MDA-MB-231 cells (B).

**Figure 4:**
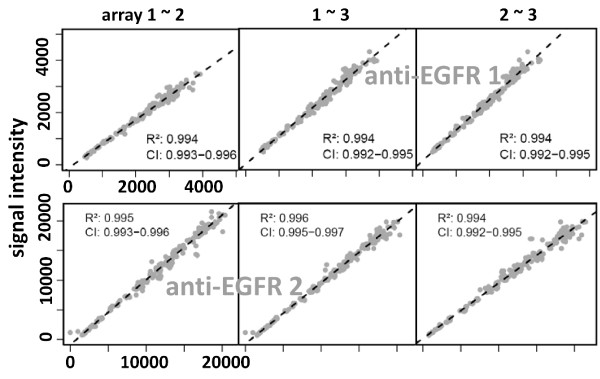
**Technical robustness of RPPA based knockdown detection**. Three replicate arrays with EGFR knockdown samples were probed with two different antibodies targeting EGFR (anti-EGFR 1 and -2). The plots show the correlation analysis for the different combinations and provide a Pearson correlation coefficient (R2) for the different comparisons.

To compare the knockdown quantification capacity of the two anti-EGFR antibodies on RPPA, the MDA-MB-231 dilution series was spotted along with dilution series prepared from siRNA-treated samples. The knockdown efficiency was quantified as described for the Western blots. On RPPA antibody 1 detected a reduced target protein expression rate of ~60% whereas antibody 2 detected a reduced expression rate of 85%, and the latter value was also obtained by Western blot (figure 3). Furthermore, whereas on Western blot the dynamic range of both antibodies differed by a factor of two, the difference increased further to almost an order of magnitude on RPPA. Samples were printed in replicate on three different slides along with two independent dilution series were used to calculate the knockdown efficiency (Table 1). A Pearson correlation coefficient of greater than 0.99 indicated that the RPPA approach is technically robust (figure 4). To sum up, anti-EGFR antibody 2 detected the reduced abundance of EGFR in the knockdown sample at a higher rate than antibody 1, indicating that the signal obtained from antibody 1 might be obscured by weak cross reactivity with other proteins which was not noticed in the Western blot analysis. Thus, anti-EGFR antibody 2 appears to work more appropriately for RPPA applications.

**Table 1:** EGFR knockdown efficiency determined by RPPA

Antibody^a^	Calibrator^b^	Array^c^		
		
		1	2	3
**EGFR #1**	1	59%	60%	61%
		
	2	58%	58%	60%

**EGFR #2**	1	85%	85%	84%
		
	2	85%	85%	84%

^a ^Two different anti-EGFR antibodies (#1, #2) were used to quantify the EGFR knockdown.

^b ^Each subarray contained two different calibrator dilution series (1, 2) printed in parallel with biological replicates of the EGFR knockdown and controls.

^c ^Three different subarrays (1-3) were printed and analyzed. Quantitative readout calculated in percent based on signals from control transfections with non-targeting siRNAs.

### Signal intensity is independent from knockdown quantification rate

**Figure 5:**
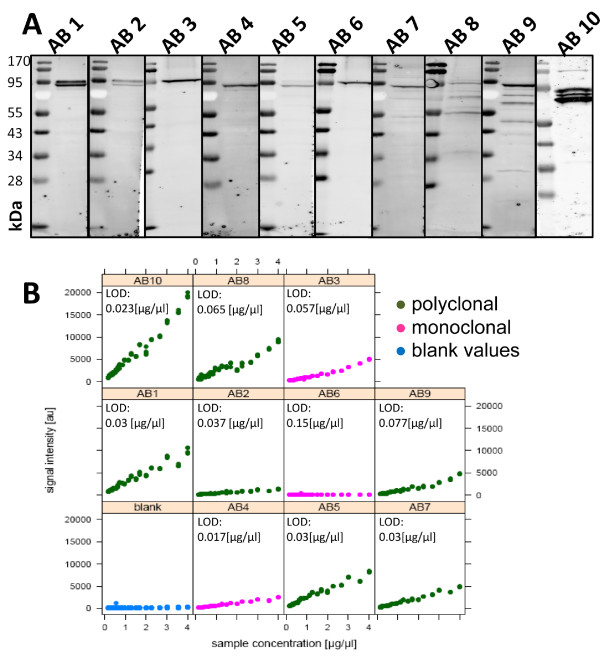
**Target specificity and signal/concentration ratio of STAT3 antibodies**. Ten antibodies targeting STAT3 were tested on lysates of MDA-MB-231 cells by Western blot. Antibodies 1-6 showed high specificity whereas antibodies 7-10 detected additional bands of lower molecular weight (A). RPPA signal dynamics of STAT3 antibodies probed on lysates of MDA-MB-231 cells (B).

To assess a general applicability of RNAi for RPPA antibody validation the approach was validated on a larger set of antibodies. In detail, ten commercially available antibodies against STAT3 obtained from different suppliers were compared (Additional file 1 Table S1). The set of ten anti-STAT3 antibodies was selected based defined applications; only antibodies of the IgG type, generated in mouse or rabbit, recommended for Western blot as well as immuno-precipitation, and advertised as being specific for the detection of the human STAT3 protein were chosen. While anti-STAT3 antibodies 1 and 2 detected a double band (figure 5A) on Western blot, antibodies 3-6 revealed a single band of the expected size. Antibody 7 produced a predominant specific band and several weak but unspecific bands. Antibodies 8-10 were considered as unspecific as they detected several additional and obviously unspecific bands by Western blot.

**Figure 6:**
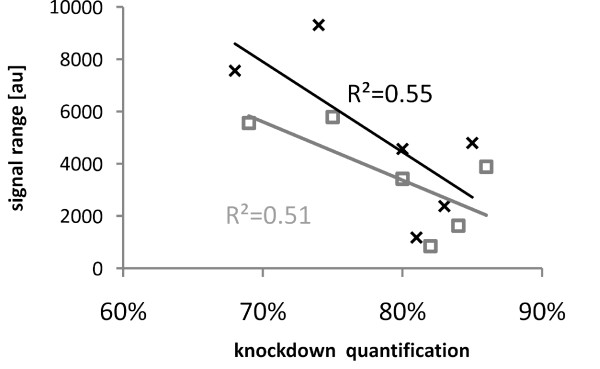
**Comparing antibody signal dynamics and quantitative readout after STAT3 silencing**. The RPPA signal range of six highly specific anti-STAT3 antibodies was correlated with the quantitative readout obtained for each of the respective antibodies when applied for the RPPA analysis of STAT3-silencing (R^2^). The data set was calculated twice by using each of the two independent calibrator dilution series (black and grey). The correlation coefficient was calculated using MS Excel, the linear regression line was added.

In the EGFR-antibody example, the EGFR-knockdown was recognized less efficiently by the weaker antibody. Thus, the question whether the signal intensity range could possibly be related to the RPPA outcome was addressed experimentally using the set of ten STAT3 antibodies. Figure 5B summarizes the RPPA signal dynamics of all ten anti-STAT3 antibodies. For nine antibodies, a strict correlation between signal intensity and total protein concentration was observed. Signals from antibody 6 were comparable to the background signals obtained in control incubations without primary antibody. Signal ratios were different for the remaining set of nine anti-STAT3 antibodies, and none of the antibodies reached signal saturation. In general, rabbit polyclonal antibodies displayed significantly stronger signals than mouse monoclonal antibodies. A linear correlation between signal intensity and protein concentration was observed for the nine anti-STAT3 antibodies over a >20-fold concentration range. To demonstrate that RPPA signals are target protein-specific, a STAT3-knockdown was quantified by probing each of the anti-STAT3 antibodies on separate arrays. The concentration of STAT3 was calculated for knockdown samples and controls as described for EGFR and table 2 summarizes the data. Those antibodies showing multiple bands corresponding to the molecular weight of STAT3 (AB 8-10) on Western blot detected the knockdown at a rate between 32 and 57% while the specific antibodies 1-5 identified the STAT3 knockdown at a rate ranging between 69 and 86%. Moreover, antibody 7, which shows additional faint low molecular weight bands by Western blot, quantified the STAT3 knockdown at a rate of 80%. Finally, anti-STAT3 antibodies 1-5 and 7 detected the STAT3 knockdown at a rate > 69%. Knockdown detection and RPPA signal dynamics were correlated in Figure 6. The correlation coefficient indicates that the signal dynamics and specificity are not directly connected with each other; antibody with weak signaling dynamics can recognize their target protein with high specificity.

**Table 2:** Quantification of STAT3 knockdown and determination of STAT3 detection limits

Antibody	Knockdown efficiency		LOD [μg/μl]^b^	
	**1**^**a**^	**2**^**a**^	**1**^**a**^	**2**^**a**^

AB1	75%	74%	0.027	0.033

AB2	82%	81%	0.035	0.038

AB3	86%	85%	0.057	0.057

AB4	84%	83%	0.016	0.018

AB5	69%	68%	0.029	0.031

AB6	-2%	-3%	0.173	0.134

AB7	80%	80%	0.031	0.030

AB8	32%	33%	0.073	0.057

AB9	57%	56%	0.077	0.077

AB10	53%	52%	0.023	0.023

^a ^Two different MDA-MB231 dilution series (1, 2) were used to calculate the percentage of the STAT3 knockdown.

^b ^The limit of detection (LOD) was calculated for AB1-10 and is expressed as μg/μl MDA-MB231 lysate.

### Introducing the detection limit as criterion for RPPA antibody validation

Four out of ten anti-STAT3 antibodies (AB 2, 3, 4, and 7) detected the knockdown of STAT3 at a rate ranging between 80 - 86%, and at a rate comparable to that detected by Western blot. Next, the detection limit was determined to identify the most sensitive antibody. For this, the median signal intensity as well as the median absolute deviation (MAD) was calculated from the readings of blank spots for each of the four anti-STAT3 antibodies. Next, the limit of detection was calculated from the median of the blank spots by adding five times the MAD of the respective antibody (median signal intensities for blank spots ranged from 8 [au] for antibody 4 to 200 [au] for antibody 8). Next, a linear regression model was fit on the serial dilutions of the positive controls to calculate the detection limit of each antibody (Table 2). Anti-STAT3 antibody 4 still detected endogenous STAT3 at a total protein concentration when the MDA-MB 231 cell lysate was diluted down to 0.017 μg/μl and was identified as the most sensitive antibody. Antibody 1 and antibody 7 were ranked second and detected endogenous STAT3 down to a total protein concentration of 0.030 μg/μl MDA-MB 231 cell lysate, and antibody 3 required almost twice the amount of total protein and was the least sensitive antibody.

### Recognizing highly homologous proteins

**Figure 7:**
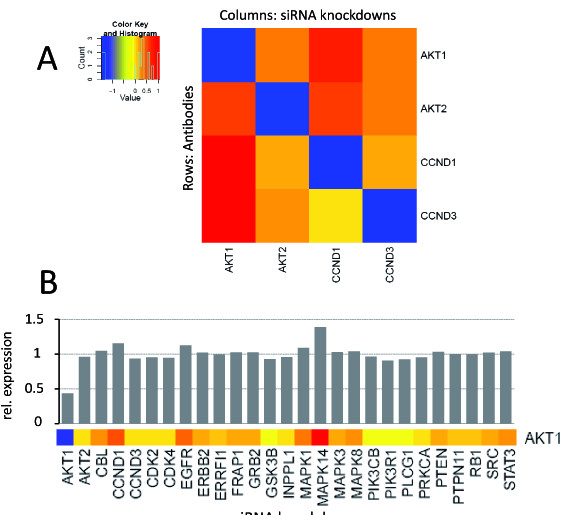
**Determination of isoform-specificity by RNAi and its application for network analysis**. AKT 1 and AKT 2 as well as cyclin family members CCND1 and CCND3 were knocked down individually and the protein levels were then detected specifically by RPPA. Low protein levels are represented in blue and high levels in red (A). The 27 indicated genes were knocked down individually with specific siRNAs and the effect on AKT1 protein levels was measured using an isoform-specific antibody. The heatmap section shows a reduced (blue) or elevated expression (red) of AKT1 in response to a targeted knockdown of the 27 genes (B).

**Figure 8:**
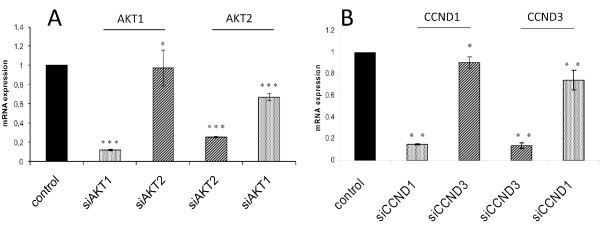
**Knockdown validation using quantitative real time PCR**. Knockdown of AKT1 and AKT2 as well as CCND1 and CCND1 mRNAs upon siRNA transfection into MDA-MB-231 cells was measured by quantitative real-time PCR. Respective siRNAs applied in the transfection are indicated in relation to the relative mRNA expression levels (values normalized to non-targeting controls ACTB and HPRT mRNAs).

To demonstrate that the RNAi-based strategy of antibody validation is also suitable for the identification of isoform-specific antibodies, two pairs of highly homologous target proteins were chosen to proof this in principle. In detail, two members of the cyclin family, CCND1 (cyclinD1) and CCND3 (cyclinD3) as well as AKT1 and AKT2 were knocked-down by RNAi and detected with antibodies supposed to recognize specifically the corresponding isoforms. Beforehand, the Needleman-Wunsch algorithm was applied to determine the degrees of similarity as well as of identity: The cyclins show an identity ratio of 51.7% and a similarity ratio of 67% whereas the AKT proteins are more closely related and revealed 81.1% sequence identity and 91.9% similarity. Silencing was performed for 48 h and the experimental data were analyzed by RPPA and summarized as ordered heatmap (figure 7A). Thus, the data demonstrated a specific detection of all four proteins as indicated by the blue color. All four antibodies distinctly detected the knockdown of the respective target protein, and none of them revealed cross reactivity with its homologous counterpart. Even the highly related proteins AKT1 and AKT2 were detected specifically. Figure 8 shows data from the corresponding qRT-PCR analysis confirming a downregulation of the targeted transcripts (figure 7A).

### Benefits of RNAi-based antibody validation for clinical and basic research

As shown for a set of only four targets proteins, the use of highly specific RPPA-validated antibodies in combination with the targeted knockdown of selected proteins provided quantitative data which were useful to dissect regulatory edges in signaling networks. Thus, proteomic data can in principle aid network reconstruction and the elucidation of coregulation and codependencies. To illustrate the potential of this approach, the impact of a knockdown of 27 different proteins on the AKT1 protein level is shown in figure 7B. First, the AKT1 antibody clearly identified the AKT1 knockdown sample. The abundance of AKT1 was not influenced by the majority of siRNA-targeted proteins including also the knockdown of AKT2 which was already seen in figure 7A. In contrast, the knockdown of MAPK14 resulted in a strong increase of AKT1 abundance on the protein level. This observation is presumably well in line with biology as the opposite event, the AKT-mediated down regulation of p38 signaling was reported to occur in endothelial cells [22].

## Discussion

Several studies presented antibody validation methods for use in immune-histochemistry [23,24] stating that thorough testing of antibodies is mandatory [24]. Accordingly, we have demonstrated here that using Western blot approaches for antibody characterization is not sufficient for the identification of RPPA-suitable antibodies. In contrast, a combination of targeting those proteins which are presumably recognized by the antibodies of choice by siRNA permitted a thorough validation of antibody specificity.

All antibodies tested in this study were commercially available and were obtained from different suppliers. The knockdown of STAT3 or EGFR by RPPA was generally detected with high specificity but differing quantitative readout. Monoclonal antibodies are directed against single epitopes, and the peptide sequence recognized by a specific antibody might be inaccessible under the native conditions of the RPPA. Furthermore, highly specific monoclonal Western blot antibodies do not necessarily work equally well on RPPA. For example, although STAT3 antibody 5 detected a single band on Western blot, the STAT3 knockdown was quantified at a rate of only 69% whereas other antibodies determined the STAT3-knockdown in the range of 80-85%. On the other hand, anti-STAT3 antibody 7 revealed cross reactivity on Western blot but demonstrated high specificity on RPPA with a STAT3-knockdown rate of 80%. Western blot and RPPA are for several reasons not directly comparable. First of all, different buffers were used for sample preparation which might influence the display of protein epitopes. Moreover, unpurified and crude samples are exposed to antibody-based detection on RPPA whereas the SDS-PAGE-based sample separation might remove certain impurities. For example, cellular debris does not enter the separation gel whereas degraded protein leave the gel quickly and are potentially eluted into the SDS-PAGE running buffer. Besides that, the velocity of electrophoretic transfer during the Western blot procedure depends on the charge as well as the size of a certain protein, e.g. small proteins are transferred faster and might be eluted into the Western blot transfer buffer whereas very large proteins might remain in the gel. In conclusion, the exact quantification of RNAi-based knockdown in biological samples presents a reliable strategy for the characterization of antibody properties for RPPA applications. The chances to detect a false negative result after performing a silencing experiment were considered as unlikely especially since appropriate control experiments were performed and analyzed in parallel.

RPPA are customized assays and are used in different experimental setups with respect to sample preparation and target protein detection. Loebke and colleagues used near-infrared (NIR) labeled secondary antibodies for signal detection on RPPA and employed a native lysis protocol to avoid protein denaturation [11]. Tibes and colleagues printed denatured samples and visualized the protein concentration using enzyme-based signal amplification with colorimetric readout (DAKO cytomation) [13], while Berg and colleagues collected samples from formalin fixed samples and detect the proteins by ECL [6]. Even other combinations of samples and detection methods can be envisioned. The diversity in the experimental setup of RPPA applications requires a proper validation of antibodies for a specific protocol and the biological context. The approach presented here will most likely also be useful for other RPPA setups since it allows the assessment of antibody specificity in a certain biological context and independently from sample preparation protocol and detection method. Furthermore, the strategy presented here could also be applied to the characterization of alternative protein binders such as Darpins (ankyrin repeat proteins) [25]. Quantification of protein activation or inhibition within a particular signaling network is another promising RPPA application which requires availability of phospho-specific antibodies [21] and demands also tools for efficient antibody validation. Silencing of endogenous proteins is superior over using a spike-in recovery of recombinant target proteins. First, the conformation of endogenous proteins might be different from the recombinant proteins due to changes of protein folding, aberrant disulfide bridge formation, and the lack of suitable posttranslational modification or the introduction of new modifications. Nevertheless, RPPA can be used to quantify robustly the impact of a particular treatment on target protein regulation for both directions, up- as well as downregulation, especially when signal detection is performed with fluorescent dyes in the NIR range since rarely signal saturation is reached. Antibodies detecting multiple bands on Western blot are not necessarily unspecific but might recognize proteins regulated by post-translational modification, breakdown products, or splice variants. In such cases, the method introduced here will provide evidence for the specificity of an antibody that could not be clarified otherwise.

The isoform-specific detection of proteins within signaling networks is required for the unambiguous identification of an individual contribution to highly regulated signaling processes. Since isoforms of proteins frequently express highly homologues peptide sequences, antibody specificity is of utmost importance for this application. Our data suggest that the siRNA-mediated antibody validation approach is highly capable to test the specificity of antibodies and especially useful to characterize antibodies which presumably recognize a particular isoform. On the other hand, this approach can thus provide key information on the regulation of protein networks to unravel signaling networks. Therefore, this validation method will extend the practical applicability of the RPPA technology in the field of systems biology and will open up new prospects for proteome research.

## Conclusions

We have introduced a new antibody validation approach which is based on a targeted knockdown strategy and subsequent quantification of the proteins of interest to quantify the difference between endogenous and residual target protein abundance by RPPA. Employing a targeted knockdown strategy we have demonstrated the potential of our approach to identify and validate antibodies that are able to distinguish highly homologous isoforms and to generate systems level information useful for protein network analysis.

## Methods

### Cell culture and siRNA transfection

The human breast cancer cell line MDA-MB-231 (HTB-26) was obtained from ATCC (Manassas, VA, USA). Cells were cultured in Leibovitz's L-15 medium (Sigma, St Louis, MO, USA) and supplemented with 50 U/mL penicillin, 50 μg/mL streptomycin sulphate, 1% non-essential amino acids, 10% fetal bovine serum (Gibco-BRL, Bethesda, MD, USA) and 3 g/L sodium bicarbonate (AppliChem, Darmstadt, Germany). MDA-MB-231 cells were seeded in 6-well format at 2 × 10^5 ^cells/well and cultivated for 24 h at 37°C and 5% CO_2_. Prior to transfection, medium was replaced with antibiotics-free medium. Cells were transfected with siRNAs targeting *EGFR*, *STAT3*, *AKT1, AKT2*, *CCND1*, and *CCND3 *purchased from Dharmacon (Lafayette, CO, USA). For each gene, four individual siRNAs were pooled (Additional file 2 Table S2). Allstar siRNA (Qiagen, Hilden, Germany) was used as non-silencing control. siRNAs were transfected by using Lipofectamine 2000 (Invitrogen, CA, USA) at a final concentration of 20 nM and cells were harvested after 48 h of further incubation.

### Quantitative real time PCR

Total RNA from MDA-MB-231 was extracted by using the RNeasy Mini Kit (Qiagen, Hilden, Germany). cDNA was generated with the Revert Aid H Minus First Strand cDNA Synthesis Kit (Fermentas St. Leon-Rot, Germany) using 0.5 μg oligo (dT) primers with 2 μl of total RNA. mRNA quantification of the target and housekeeping genes *ACTB *and *HPRT *was performed with ABI Prism 7900HT Sequence Detection System (Applied Biosystems, Weiterstadt, Germany) using probes of the Universal Probe Library (Roche, Penzberg, Germany). Primer sequences and matching UPL probe numbers are given in Additional file 3 Table S3. Data was analyzed with the *ddCt *algorithm (Bioconductor *package*) and mRNA levels were normalized to the level of the housekeeping genes.

### Cell lysis and Western blotting

Samples were trypsinized from the cell culture dish; cells were pelleted and stored at minus 80°C until lysis. The lysis buffer (MPER, Thermo Scientific) was supplemented with protease inhibitor (miniComplete, Roche). Cells were suspendend in 25 μl of lysis buffer and lysed for 20 min at 4°C on a rotating wheel. After centrifugation, approximately 25 μl of protein extract was obtained from a single well. Protein concentration was determined using a modified BCA protein assay [26] (Pierce, Bonn, Germany). Samples were mixed with standard protein loading buffer (2×) (Roth, Karlsruhe, Gemany) and heated for 5 min at 95°C. Proteins were subjected to electrophoretic separation at 125 mV for 75 min and transferred in a semi-dry approach on PVDF membrane for 60 min at 25 mV. The membrane was blocked for 60 min in Odyssey blocking buffer/PBS (50% v/v). Detection antibodies were diluted 1:1000 into blocking buffer and incubated on the membrane over night at 4°C. Unbound primary antibody was removed by carrying out 5 washing cycles with 0.1% (v/v) Tween/PBS. Secondary NIR-dye labeled antibodies were diluted 1:8000 in 0.05% (v/v) Tween/PBS and incubated in the membrane for 1 hour at RT. For signal detection, membranes were scanned using the Odyssey imaging system (LI-COR Biosciences, Lincoln, USA).

### Reverse phase protein array

The total protein concentration was adjusted to 3 μg/μl for samples >3.3 μg/μl, and left unchanged for concentrations below this value. All sampled were transferred to a 384-well plate. Tween was added to result in a final concentration of 0.05% (w/v). Using an Aushon 2470 microarrayer (Billerica, MA) equipped with 185 μm solid pins approximately 1.6 nl sample was delivered per spot onto nitrocellulose coated slides (Oncyte Avid, Grace Bio-labs, Bend, OR, USA). Samples were printed in three subarrays per slide and in four replicate spots. Slides were stored at -20°C. To estimate the total protein concentration per spot a slide from each print run was stained with Fast Green FCF (Sigma-Aldrich, Steinheim, Germany) as described before [11]. Slides were mounted in a customized incubation chamber (Metecon, Mannheim, Germany) to form three individual incubation chambers on top of each subarray. Prior to antibody staining, arrays were blocked for 1 h at RT with 50% (v/v) Odyssey blocking buffer in PBS. Slides were then incubated with target protein specific antibodies diluted 1:300 in blocking buffer at 4°C over night. After washing the slides, secondary antibodies were diluted 1:8000 in PBS with 0.05% Tween and incubated for 1 h at RT. Finally, slides were washed, the chamber was unmounted and air dried.

### Data analysis

Slides were scanned at 21 μm resolution on an Odyssey scanner (LI-COR, Lincoln, NB, USA) and images were saved as 16 bit gray scale TIF files. Analysis of the TIF files was performed using GenePix Pro 5.0 microarray analysis software (Molecular Devices, Silicon Valley, CA, USA). Data analysis was done using R and the package RPPanalyzer [27]. For each antibody the logged mean pixel intensity of a single spot was subtracted by the corresponding Fast Green FCF signal to normalize for the total protein immobilized per spot. Knockdown recovery was calculated by fitting a linear model on the calibrator dilution series, the virtual concentration of the knockdown samples, and the control transfection samples was calculated using the coefficients of the linear model.

## Competing interests

The authors declare that they have no competing interests.

## Authors' contributions

HAM and OS had the idea to use RNAi for antibody validation; HAM, OS, SU and UK planned the experiments; HAM and SU carried out the experiments; CS was responsible for automated siRNA transfection and microarray printing; HAM performed data analysis; SW and UK were responsible for lab organization, equipment, and resources; HAM and SU wrote the first draft of the manuscript; SW, OS, and UK critically reviewed the content of the manuscript before submission. All authors read and approved the final manuscript.

## Supplementary Material

Additional file 1 **Table S1. Antibodies used for RPPA detection**. Table summarizes information provided by antibody suppliers; antibody names, order number, supplier, host used to produce the antibody, antibody type, epitope/domain recognized (if known), certified applications, species specificity. Click here for file

Additional file 2 **Table S2. Sequences of siRNAs targeting *AKT1, AKT2*, *CCND1 *and *CCND3***. Table lists the exact sequence of the four siRNAs which were used per targeted transcript. Click here for file

Additional file 3 **Table S3. Primer Sequences used in TaqMan assays**. Table lists 5' and 3' specific primers used in qPCR reactions with reference to the UPL probe number. Click here for file
