# Supplementary material for: RNAi-based validation of antibodies for reverse phase protein arrays
Source: Proteome Sci. 2010 Dec 23;8:69. doi: 10.1186/1477-5956-8-69 (PMC3022873; doi:10.1186/1477-5956-8-69)
Supplement: Additional file 1 — Table S1. Antibodies used for RPPA detection. Table summarizes information provided by antibody suppliers; antibody names, order number, supplier, host used to produce the antibody, antibody type, epitope/domain recognized (if known), certified applications, species specificity. [file 1477-5956-8-69-S1.PDF]

Supplementary table 1: Description of antibodies

Antibody	Order number	Supplier	Clonal status	Host	(advertised) MW [kDa]	Epitope	Applications	Reactivity
STAT3-AB1	9132	Cell Signaling	poly	rabbit	79; 86	NA	WB, IP, IHC, ChiP	H, M, R
STAT3-AB2	569388	Calbiochem	poly	rabbit	91?	C-terminus	WB, IP, ICH	H, M, R
STAT3-AB3	4904	Cell Signaling	mono	rabbit	79; 86	C-terminus	WB, IP, IHC, ChiP	H, M, R, Mk
STAT3-AB4	610190	Becton-Dickinson	mono	mouse	92	N-terminus	WB, IP	H, D, Rat, M, Chick
STAT3-AB5	ab3162	Millipore	poly	rabbit	88	C-terminus	WB, IP	H, M, Rat
STAT3-AB6	9139	Cell Signaling	mono	mouse	79; 86	NA	WB, IP, ICH-P, IF-IC, F, ChiP	H, M, R, Mk
STAT3-AB7	sc-483	Santa-Cruz	poly	rabbit	86 (β);91 (α)	internal	WB, IP, IF, ELISA, ChiP, GS	H, M, Rat
STAT3-AB8	06-569	Millipore	poly	rabbit	92	aa 688-722	WB, IP, ChiP	H, M, Rat
STAT3-AB9	sc-7179	Santa-Cruz	poly	rabbit	86 (β);91 (α)	aa 50-240	WB, IP, IF, ICH, ELISA, ChiP, GS	H, M, Rat, Z, F
STAT3-AB10	sc-482	Santa-Cruz	poly	rabbit	86 (β);91 (α)	C-terminus	WB, IP, IF, ELISA, Chip, GS	H, M, Rat, F
EGFR-AB1	sc-03	Santa-Cruz	poly	rabbit	NA	C-terminus	WB, IP, IF, ICH, ELISA	H, M, Rat
EGFR-AB2	2646	Cell Signaling	mono	rabbit	175	cytoplasmic domain	WB, IP	H, M, R, Mk
AKT1	610860	Becton-Dickinson	mono	mouse	59	full length	WB, IF	H, D, M, Rat
AKT2	3063	Cell Signaling	mono	rabbit	60	full length	WB, IP	H, M, R, Mk
CDK2	C5223	Sigma	poly	rabbit	33	aa 287-298	WB, IP	H
CDK4	2341-1	Epitomics	mono	rabbit	34	N-terminus	WB, FC, IP	H
