# Supplementary material for: RNAi-based validation of antibodies for reverse phase protein arrays
Source: Proteome Sci. 2010 Dec 23;8:69. doi: 10.1186/1477-5956-8-69 (PMC3022873; doi:10.1186/1477-5956-8-69)
Supplement: Additional file 2 — Table S2. Sequences of siRNAs targeting AKT1, AKT2, CCND1 and CCND3. Table lists the exact sequence of the four siRNAs which were used per targeted transcript. [file 1477-5956-8-69-S2.DOC]

**Supplementary Table 2. Sequences of siRNAs targeting *AKT1, AKT2*, *CCND1* and *CCND3.***

| **Gene** | **siRNA no.** | **Sequence** |
| --- | --- | --- |
| *AKT1* | 1 | GACAAGGACGGGCACAUUAUU |
|  | 2 | GGACAAGGACGGGCACAUUUU |
|  | 3 | GCUACUUCCUCCUCAAGAAUU |
|  | 4 | GACCGCCUCUGCUUUGUCAUU |
| *AKT2* | 1 | ACACAAGGUACUUCGAUGA |
|  | 2 | GCAAGGCACGGGCUAAAGU |
|  | 3 | GUGAAUACAUCAAGACCUG |
|  | 4 | CAUGAAUGACUUCGACUAU |

| *CCND1* | 1 | GUUCGUGGCCUCUAAGAUGUU |
| --- | --- | --- |
|  | 2 | CCGAGAAGCUGCAUCUAUU |
|  | 3 | GAACAGAAGUGCGAGGAGGUU |
|  | 4 | ACAACUUCCUGUCCUACUAUU |
| *CCND3* | 1 | GGACCUGGCUGCUGUGAUU |
|  | 2 | UGCGGAAGAUGCUGGCUUA |
|  | 3 | GAGCUGCUGUGUUGCGAAG |
|  | 4 | GAUCGAAGCUGCACUCAGG |
