# Supplementary material for: RNAi-based validation of antibodies for reverse phase protein arrays
Source: Proteome Sci. 2010 Dec 23;8:69. doi: 10.1186/1477-5956-8-69 (PMC3022873; doi:10.1186/1477-5956-8-69)
Supplement: Additional file 3 — Table S3. Primer Sequences used in TaqMan assays. Table lists 5' and 3' specific primers used in qPCR reactions with reference to the UPL probe number. [file 1477-5956-8-69-S3.DOC]

**Supplementary Table 3. Primer Sequences and Universal Probe Library (UPL) probe number used for qRT-PCR**.

| **Gene** | **Primer left** | **Primer right** | **UPL probe** |
| --- | --- | --- | --- |
| ***ACTB*** | CCAACCGCGAGAAGATGA | CCAGAGGCGTACAGGGATAGC | #64 |
| ***HPRT1*** | TGACCTTGATTTATTTTGCATACC | CGAGCAAGACGTTCAGTCCT | #73 |
| ***AKT1*** | GCAGCACGTGTACGAGAAGA | GGTGTCAGTCTCCGACGTG | #45 |
| ***AKT2*** | CCATGAATGAGGTGTCTGTCA | GGCCTCCAGGTCTTGATGTA | #36 |
| ***CCND1*** | CGGAATCGTGAGGATCGTA | GACCTCCTCCTCGCACTTCT | #67 |
| ***CCND3*** | GCTTACTGGATGCTGGAGGTA | AAGACAGGTAGCGATCCAGGT | #68 |
